# Incidence and survival of pediatric low-grade gliomas in Switzerland

**DOI:** 10.1007/s12519-026-01085-8

**Published:** 2026-09-12

**Authors:** Ladina Greuter, Eleftheria Michalopoulou, Shelagh Redmond, Nicolas V. der Weid, Maria Licci, Johanna Wyss, Raphael Guzman, Jehuda Soleman

**Affiliations:** 1 Department of Neurosurgery, University Hospital of Basel, Basel, Switzerland; 2 Division of Pediatric Neurosurgery, University Children’s Hospital of Basel, Basel, Switzerland; 3 Childhood Cancer Registry, Institute of Social and Preventive Medicine, Faculty of Medicine, University of Bern, Bern, Switzerland; 4 Division of Pediatric Oncology, University Children’s Hospital of Basel, Basel, Switzerland; 5 Faculty of Medicine, University of Basel, Basel, Switzerland

**Keywords:** Children, Epidemiology, Low-grade glioma, Neurosurgery, Pediatric

## Abstract

**Background:**

Brain tumors are the most common solid cancers occurring in children, with pediatric low-grade gliomas (pLGG) being the most frequent central nervous system (CNS) tumors in children. The overall survival of pLGG is excellent, with a ten-year survival of 85%–96%. Prognostic factors include age, gender, anatomical location, and histological and molecular subtype. Despite the excellent survival rates, children suffering from pLGG often have a diminished quality of life due to functional or neurologic deficits either caused by the primary tumor or as a side effect of their treatment. In this study, we describe the epidemiology of pLGG in Switzerland.

**Methods:**

The target population comprised children aged 0–17 years, diagnosed with histologically verified pLGG during 1990–2019 while residing in Switzerland. We retrieved the data from the Swiss Childhood Cancer Registry (ChCR). We calculated crude incidence rates per 100,000 person-years and observed survival (OS) using the Kaplan-Meier estimator (non-parametric univariable analysis). We estimated OS separately according to age histological subgroup and treatment group.

**Results:**

We included 591 pLGG during 1990–2019 in Switzerland. Most pLGG were diagnosed at the age of 2–11 years (*n* = 352, 59.6%). Around half of pLGG were supratentorial located (*n* = 286, 48.4%), while the other half (*n* = 266, 45.0%) occurred infratentorial. Most pLGG (82.0%) were World Health Organization (WHO) grade 1. The vast majority of pLGG (*n* = 501, 84.8%) were treated with surgery, either alone (*n* = 441, 74.6%) or in combination with other treatments. The lowest 5- and 10-year survival was observed in children who received surgery followed by radiotherapy and/or chemotherapy (40% and 20%). Astrocytoma excluding pilocytic astrocytoma had a significantly lower 5-year, 10-year and total survival (98.8%, 85.8%, and 82.1%) compared to the other histopathological subtypes (*P* = 0.024).

**Conclusions:**

This is the first report on the incidence and survival of children with pLGG in Switzerland. Children with pLGG have an excellent long-term survival rate of over 90%.

## Introduction

Brain tumors are the most common solid cancers occurring in children, with low-grade pediatric gliomas (pLGG) being the most frequent central nervous system (CNS) tumors [1–4]. The pLGG comprises a heterogeneous group of tumors classified as World Health Organization (WHO) grade 1 or 2. It is reported that approximately (65%) of pLGGs are pilocytic astrocytoma (WHO 1) [4, 5]. While surgery is the main therapy for pLGG, further treatment is required in case of an incomplete resection due to their eloquent anatomical location or in case of a recurrence [6]. Adjuvant treatment conventionally included chemotherapy, sometimes radiotherapy. While chemotherapy remains the standard frontline therapy for most pLGG that recur after surgery, targeted inhibitors such as mitogen-activated protein kinase (MEK) and/or BRAF inhibitors have shown promising outcomes for relapsed tumors [4, 7–9]. Radiotherapy has generally been reserved for recurrent cases in the treatment of pLGG due to long-term toxicity, while it used to be a mainstay of therapy [10, 11]. The overall survival of pLGG is excellent, with a 10-year survival rate of 85%–96% [4, 5, 12, 13]. Prognostic factors influencing survival include age, gender, anatomical location, and histopathological and molecular subtype [14]. Despite the excellent survival rates, children suffering from pLGG often have a diminished quality of life due to functional or neurologic deficits either caused by the primary tumor or as a side effect of their treatment [4, 15], resulting in a high burden of long-term morbidity [4, 16]. Understanding the epidemiology of pLGG is important to estimate the long-term outcome for these patients and the public health impact [17]. In Germany, an increase in the incidence of pLGG has been observed over the last two decades from 0.94 to 2.12 per 100,000 children per year [18]. A similar development was observed in North America with geographic differences between the Northeast and Midwest [14].

No current study is available on the epidemiology of pLGG in Switzerland. National databases can play an essential role in conducting such epidemiological studies by collecting cancer data. In the US, the Surveillance, Epidemiology, and End Results Program (SEER) covers around half of the population’s information about cancer incidence and outcomes [17]. In Switzerland, the Childhood Cancer Registry (ChCR) is a comparable national database for cancer patients diagnosed at the age of 0–19 years. It was founded in the 1970s and all pediatric oncology centers report directly to this database [16, 19], making it a real population-based registry. In this study, we describe the epidemiology of pLGG in Switzerland, using national cancer registry data. Specifically, we report on the incidence and observed survival (OS) of pLGG and look for potential determinants of event-free survival (EFS).

## Methods

### Study population

The target population comprised children (< 18 years) diagnosed with pLGG between 1990 and 2019 in Switzerland. Based on our predefined study protocol, we selected 2019 as the follow-up cutoff to ensure data completeness and accuracy, given potential delays in fully entering all information into the database. This study only included data from the ChCR with histopathologically verified pLGG. The ChCR is estimated to include over 90% of all cancer cases diagnosed in Switzerland for children younger than 16 years, whereas registration of cancer in adolescents older than 16 years at diagnosis has been less complete, due to the possibility of these children being treated in adult cancer centers [19, 20]. No data were acquired if the parents vetoed their child’s registration in the ChCR, despite anonymous collection. However, the veto rate was low at approximately 0.5%. The ChCR validated data by receiving original pathology, surgery, and treatment or follow-up reports, as well as by submitting information on standardized forms that included individual treatment modalities.

The retrieved data on histopathologically verified pLGG included information on the date of diagnosis (set as timepoint when histopathology was achieved), age at diagnosis (grouped as 0–1, 2–11, 12–15, and 16–17 years), sex (male, female), and geographic region of diagnosis according to language (German-, French-, or Italian-speaking areas). We additionally received data on the WHO grade (1, 2, or unknown), anatomical localization (supratentorial, infratentorial, spinal cord, multifocal, or unknown), and histopathological subgroup, which was coded according to the third version of the International Classification of Childhood Cancers (ICCC3) [21]. The histopathological subgroups comprised astrocytoma (not pilocytic), pilocytic astrocytoma, other specified and unspecified intracranial and intraspinal neoplasms (neuronal and mixed neuronal-glial tumors), and other gliomas (oligodendrogliomas, mixed and unspecified gliomas, neuroepithelial glial tumors of uncertain origin). We also collected data on treatments during the first year after pLGG diagnosis and on treating hospitals, including Swiss Pediatric Oncology Group (SPOG) clinics and non-SPOG clinics. Treatments during the first year after pLGG diagnosis were grouped into surgery, chemotherapy, radiotherapy, and all the potential combinations thereof. In the case of surgery, we collected information on the extent of resection, including gross total resection (GTR), subtotal resection (STR), partial resection, and biopsy. GTR was defined as a resection with greater than 90% of the tumor mass. STR was defined as a resection of 50%–90%. Partial resection was defined as the removement of less than 50%. If available, data on underlying syndromes, such as neurofibromatosis type 1 (NF-1), tuberous sclerosis, and epilepsy, were acquired. Information on other underlying diseases was incomplete and was registered only for 13% of all the included pLGGs, which is why we did not analyze them. We acquired information on the vital status of patients at the end of the study follow-up (31st of December 2019) or the maximum available follow-up to estimate OS. We acquired the dates of progression, recurrence, and metastasis after a pLGG diagnosis to estimate EFS if those occurred. EFS was defined as the time to progression, recurrence, metastasis, or death, whichever came first. Progression was reported to the ChCR by the clinicians on a standard relapse or progression form.

### Statistical analysis

To account for incomplete registration coverage of pLGG cases diagnosed at ages 16–17, we estimated the total number of pLGG that would have been observed for this age group under nationwide coverage by reweighting the observed number of cases [22]. The weights were calculated as the ratio of the Swiss resident population to the population covered by the existing Swiss cancer registries, stratified by age and calendar years, language region, and sex. The total number of pLGG for a specific calendar period was then estimated by taking the weighted sum of the observed cases across strata over the study period.

To calculate incidence, we obtained data from the Federal Statistical Office on the Swiss resident population at year-end by sex, age (0–17 years), calendar year (1990–2019), and district (called canton) of residence. We calculated crude incidence rates per 100,000 person-years by histopathological subgroups and age groups across the entire study period (1990–2019). Crude incidence rates were calculated by dividing the aggregated estimated number of pLGG (observed for the age groups 0–1, 2–11, 12–15) by the corresponding sum of the mid-year population [22]. The mid-year population for each year was obtained by averaging the end-year populations of that year and the preceding year. The unit of incidence rate is per 100,000 children per year.

Kaplan-Meier analysis was used to calculated OS, and log-rank test was used to assess differences. Follow-up time lasted until the date of death, the latest date of available information on vital status, or the end of follow-up, whichever came first. We estimated OS separately according to age at diagnosis, 10-year calendar period of pLGG diagnosis (1990–1999, 2000–2009, 2010–2019), histopathological subgroup, treatment group, and anatomical localization.

To investigate potential determinants of EFS, we fitted a Cox model, assuming proportional hazards. Age at diagnosis, sex, calendar period of diagnosis, geographic region, anatomical localization, histopathological subgroup, WHO grade, the presence of NF-1 (yes or unknown), treating hospital and treatment group were included as covariates in the model, and estimated adjusted hazard ratios (HRs) and 95% confidence intervals (95% CIs) for the combined event of progression, recurrence, metastases, and death (whichever came first). Overall *P* values were estimated by likelihood ratio test comparing the full model, which included all the covariates mentioned above, to the model excluding each time one covariate.

We assessed 1-, 5- and 10-year survival (observed and event-free) trends for the periods 1995–2004, 2005–2014 and 2015–2019. For the most recent period (2015–2019), we estimated survival using the period monitoring scheme to get up-to-date long-term survival estimates [23]. We excluded one pLGG patient from all survival analyses, as he was diagnosed by death certificate only (DCO), meaning that the date of death was assigned to the date of diagnosis (zero follow-up time). All analyses were performed in R statistical software [24], and the survival analyses were conducted using “survival” R-package [25].

## Results

### Baseline demographics

A total of 591 pLGG patients were obtained, which account for 37.8% of all registered CNS tumors during 1990–2019 (Table 1). Out of those, 315 (53.3%) were diagnosed in males and 276 (46.7%) in females. Most pLGG were diagnosed between the ages of 2 and 11 years (*n* = 352, 59.6%). Around half of all pLGG were located supratentorial (*n* = 286, 48.4%), while the other half (*n* = 266, 45.0%) occurred infratentorial, and only a few were located either in the spinal cord (*n* = 27, 4.6%) or in multiple or unknown locations (*n* = 12, 2.0%).

**Table 1:** Baseline population characteristics

Characteristic	Observed number of pLGG (*N* = 591) ^a^
Diagnosis age (y)	
0–1	44 (7.4)
2–11	352 (59.6)
12–15	141 (23.9)
16–17	54 (9.1)
Sex	
Male	315 (53.3)
Female	276 (46.7)
Calendar period of diagnosis	
1990–1999	146 (24.7)
2000–2009	219 (37.1)
2010–2019	226 (38.2)
Language region of diagnosis	
German/Romansh	411 (69.5)
French/Italian	180 (30.5)
Anatomical localization	
Supratentorial	286 (48.4)
Infratentorial	266 (45.0)
Spinal cord	27 (4.6)
Multiple or unknown	12 (2.0)
Treating hospital	
SPOG	457 (77.3)
Non-SPOG	134 (22.7)
Underlying diseases^b^	
Yes	79 (13.4)
Not reported	512 (86.6)

Data is presented as *n* (%)

^a ^Number of histologically verified low-grade gliomas registered by the Childhood Cancer Registry (ChCR)

^b ^Underlying diseases consist of epilepsy or seizures, neurofibromatosis 1, and tuberous sclerosis

*pLGG* pediatric low-grade gliomas, *SPOG* Swiss Peadiatric Oncology Group

### Histopathology and treatment

In Table 2, over half of the patients were pilocytic astrocytomas (*n* = 344, 58.2%), followed by other specified and unspecified intracranial and intraspinal neoplasms (*n* = 116, 19.6%), and other astrocytomas, excluding pilocytic (*n* = 103, 17.4%), hence, most pLGG were of WHO grade 1 (*n* = 482, 81.5%). During the first year after diagnosis, the vast majority of pLGG (*n* = 501, 84.8%) were treated with surgery, either alone (*n* = 441, 74.6%) or in combination with other treatments (42 with chemotherapy, 11 with radiation, and seven with chemotherapy and radiation). Out of all the surgeries, 52.1% were gross total resections (*n* = 261). Only very few pLGG were treated with either chemotherapy (*n* = 19, 3.2%) or radiotherapy (*n* = 5, 0.8%) or with a combination of both (*n* = 10, 1.7%) without any surgery beforehand. The *NF-1* mutations were reported in 3.7% (*n* = 22) of our study population. Out of the 22 pLGG with confirmed NF-1, 18 (81.8%) had surgery with or without any additional treatment, which is comparable to the proportion of pLGG without confirmed *NF-1* mutations having surgery with or without any other treatment (*n* = 483, 84.9%).

**Table 2:** Observed number of pLGGs between 1990 and 2019 in Switzerland by tumor characteristics and treatments related factors

Characteristic	Observed number of pLGG (*N* = 591)
Histological subtype	
Astrocytomas (excluded pilocytic)	103 (17.4)
Pilocytic astrocytomas	344 (58.2)
Other specified and unspecified intracranial and intraspinal neoplasms ^a^	116 (19.6)
Other gliomas ^b^	28 (4.7)
WHO grade	
Grade 1	482 (81.5)
Grade 2	105 (17.8)
Unknown	4 (0.7)
NF-1	
Yes	22 (3.7)
Not reported	569 (96.3)
Treatment within the 1 y of diagnosis	
Surgery	441 (74.6)
Chemotherapy	19 (3.2)
Radiotherapy	5 (0.8)
Surgery and chemotherapy	42 (7.1)
Surgery and radiotherapy	11 (1.9)
Radiotherapy and chemotherapy	10 (1.7)
Surgery, chemotherapy and radiotherapy	7 (1.2)
Incomplete information	48 (8.1)
No treatment	8 (1.4)
Extent of resection	
Gross total	308 (52.1)
Subtotal	177 (29.9)
Biopsy only ^c^	90 (15.2)
Unclear	16 (2.7)

Data is presented as *n* (%)

^a ^Consist of neuronal and mixed neuronal-glial tumors

^b ^Consist of oligodendrogliomas, mixed and unspecified gliomas, neuroepithelial glial tumors of uncertain origin

^c ^Biopsy was not counted to the treatment group “surgery” given it only served to obtain a histopathology

*pLGGs* pediatric low-grade gliomas, *WHO* World Health Organization, *NF-1*neurofibromatosis 1

### Incidence rates

We estimated 641 pLGG diagnosed at age 0–17 years during 1990–2019 and a total incidence of 1.4 pLGG per 100,000 children per year in Switzerland, after accounting for a possibly incomplete registration coverage for pLGG diagnosed at the age of 16–17 (Table 3). Incidence was similar across the older age groups (2–11 years, 12–15 years, and 16–17 years), ranging from 1.4 to 1.5except during infancy (0–1 year), with the lowest estimated incidence of 0.9. Among the histopathological subgroups, pilocytic astrocytoma had the highest incidence of 0.8, whereas other gliomas had the lowest incidence of 0.1. Over the observed interval (1990–1999 versus 2010–2019), incidence rates increased in the infant and adolescent (12–15 years) age groups from 0.85 to 1.01, and from 1.09 to 1.54, respectively.

**Table 3:** Incidence of pLGGs per 100,000 children per year between 1990 and 2019 in Switzerland by age groups and histological subtypes

Characteristic	Number of pLGGs		IR
	Observed (*N* = 591) ^a^	Estimated (*N* = 614) ^b^	
Age groups (y)			
0–1	44 (7.4)	44 (7.2)	0.92
2–11	352 (59.6)	352 (57.3)	1.45
12–15	141 (23.9)	141 (23.0)	1.42
16–17	54 (9.1%)	77 (12.5)	1.52
Histological subtypes			
Astrocytomas (excluded pilocytic)	103 (17.4)	112 (18.2)	0.25
Pilocytic astrocytomas	344 (58.2)	352 (57.3)	0.80
Other specified and unspecified intracranial and intraspinal neoplasms ^c^	116 (19.6)	122 (19.9)	0.28
Other gliomas ^d^	28 (4.7)	29 (4.7)	0.07

^a ^Number of LGGs registered by the Childhood Cancer Registry (ChCR)

^b ^Number of LGGs that would have been observed if registration of cases in 16- to 17-year olds had been nationwide

^c ^Consist of neuronal and mixed neuronal-glial tumors

^d ^Consist of oligodendrogliomas, mixed and unspecified gliomas, neuroepithelial glial tumors of uncertain origin

*pLGGs* pediatric low-grade gliomas; *IR* incidence rate

### Observed and event-free survival

**Fig. 1:**
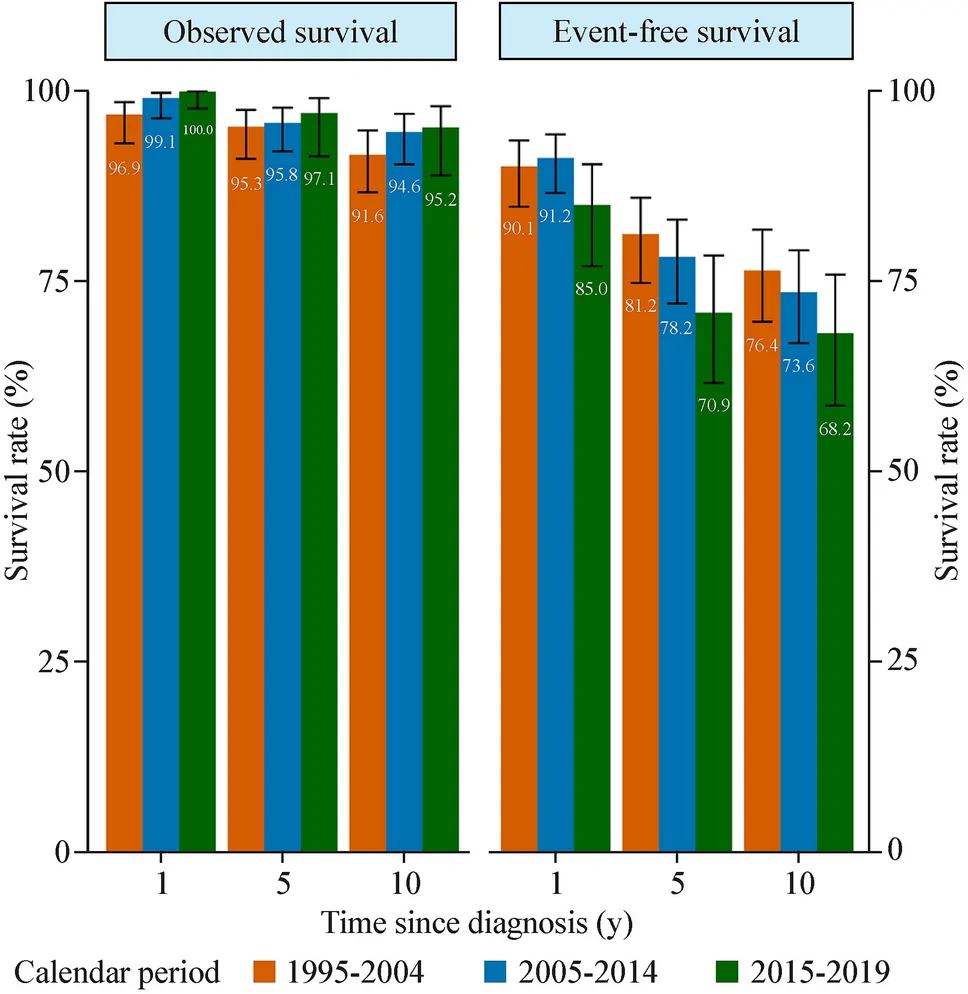
1-, 5-,10-year, and global overall survival curves by calendar period at diagnosis (y)

At the end of follow-up, 91% of children were still alive, and 26% experienced progression, recurrence, metastasis, or death. The vast majority of pLGG patients showed excellent short and long-term survival after their diagnosis. Survival increased during the observed period; for example, during 2015–2019, 5-year OS was 97.1% and 10-year survival was 95.2%, compared with 95.2% and 91.6% during 1995–2004, respectively. On the contrary, we observed a decrease in EFS over time, especially at 5- and 10-year after the pLGG diagnosis (Fig. 1).

**Fig. 2:**
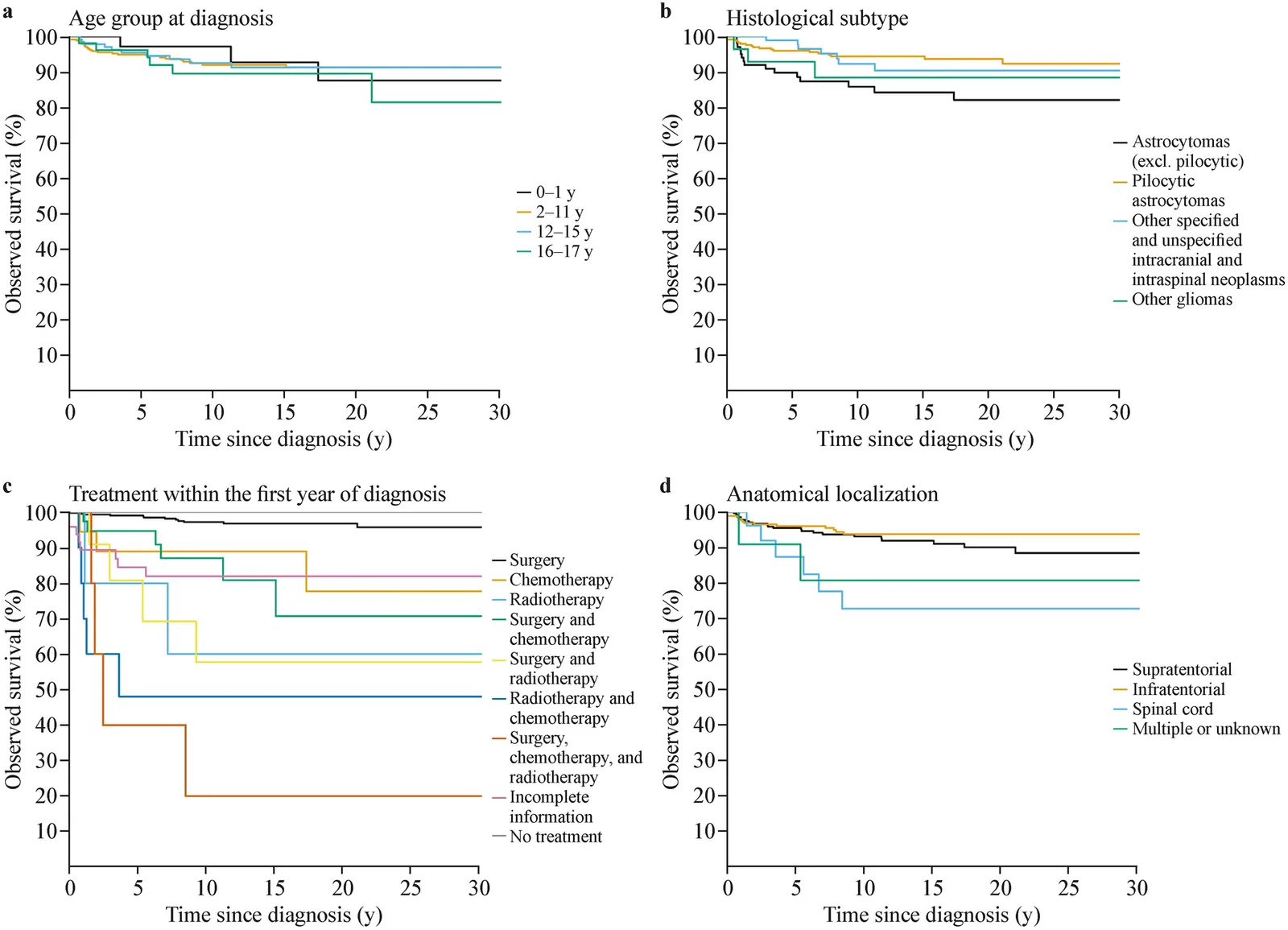
5-, 10-year, and total observed survival curves by subgroups. **a** age at diagnosis; **b** histopathological subgroup; **c** treatment type received the first year of diagnosis; **d** anatomical location

The OS appeared to be lower for the older age group compared to the rest of the age groups at the end of follow-up, but with no evidence for a significant difference in survival among the different age groups (Table 4, Fig. 2a). However, in terms of EFS after adjusting for other potential determinants, we found that the age at pLGG diagnosis was associated with the risk for recurrence, metastasis, progression, or death. The HR was lower for the age of 2–11 years (adjusted HR = 0.51, 95% CI 0.31–0.84) and 12–15 years (adjusted HR = 0.27, 95% CI 0.14–0.52) compared to the 0–1 year (Table 5). The univariable analysis showed evidence for a difference in OS by histopathological subtype. Astrocytoma, excluding pilocytic astrocytoma, showed a systematically lower OS after the first year of diagnosis compared to the other histopathological subtypes (*P* = 0.024), with the steepest decrease in the survival of this subtype being observed between two and five years of follow-up (Fig. 2b, Table 4). The highest OS at the end of follow-up was estimated for pilocytic astrocytoma (92.3%), while other intracranial neoplasms showed the highest 5-year OS (98.8%, Table 4). No associations were found between histopathological subtypes and EFS (*P* = 0.920, Table 5).

**Table 4:** Observed survival at 1, 5, and 10 y after the pLGG diagnosis and at the end of the follow-up by clinical and tumor-related characteristics

Characteristic	1 y	5 y	10 y	End of follow-up	*P* value ^a^
Age group at diagnosis (y)					
0–1	100.0 (93.0, 100.0)	97.1 (81.4, 99.6)	97.1 (81.4, 99.6)	87.6 (65.2, 96.0)	0.833
2–11	98.0 (95.8, 99.0)	94.8 (91.8, 96.8)	92.0 (88.2, 94.6)	91.3 (87.3, 94.1)	
12–15	98.6 (94.3, 99.6)	95.4 (90.1, 97.9)	92.5 (86.0, 96.1)	91.3 (84.2, 95.3)	
16–17	98.1 (87.1, 99.7)	96.2 (85.5, 99.0)	89.6 (76.6, 95.5)	81.4 (56.4, 92.9)	
Calendar period of diagnosis					
1990–1999	96.6 (92.0, 98.6)	92.5 (86.8, 95.8)	86.9 (80.3, 91.5)	86.2 (79.4, 90.8)	0.107
2000–2009	98.2 (95.2, 99.3)	95.9 (92.2, 97.8)	94.0 (89.9, 96.5)	90.5 (84.4, 94.3)	
2010–2019	99.6 (96.9, 99.9)	96.5 (92.2, 98.4)	95.4 (90.4, 97.9)	95.4 (90.4, 97.9)	
Histological subtype					
Astrocytomas (excluded pilocytic)	97.0 (91.1, 99.0)	89.8 (81.8, 94.4)	85.8 (76.7, 91.6)	82.1 (71.5, 89.1)	0.024
Pilocytic astrocytomas	98.2 (96.1, 99.2)	96.0 (93.1, 97.6)	94.3 (91.0, 96.5)	92.3 (87.3, 95.4)	
Other specified and unspecified intracranial and intraspinal neoplasms ^b^	100.0 (97.4, 100.0)	98.9 (92.7, 99.8)	92.3 (83.5, 96.5)	90.4 (80.6, 95.4)	
Other gliomas ^c^	96.4 (77.2, 99.5)	92.9 (74.3, 98.2)	88.4 (68.0, 96.2)	88.4 (68.0, 96.2)	
Treatment within 1 y of diagnosis					
Surgery	99.5 (98.2, 99.9)	99.0 (97.5, 99.6)	97.2 (94.8, 98.5)	95.7 (91.8, 97.8)	< 0.0001
Chemo	94.4 (66.6, 99.2)	88.9 (62.4, 97.1)	88.9 (62.4, 97.1)	77.8 (42.4, 92.9)	
Radio	100.0 (40.0, 100.0)	80.0 (20.4, 96.9)	60.0 (12.6, 88.2)	60.0 (12.6, 88.2)	
Surgery-chemo	100.0 (92.9, 100.0)	94.7 (80.3, 98.6)	87.1 (68.7, 95.0)	70.8 (40.7, 87.5)	
Surgery-radio	100.0 (72.7, 100.0)	80.8 (42.3, 94.9)	57.7 (22.1, 81.9)	57.7 (22.1, 81.9)	
Radio-chemo	80.0 (40.9, 94.6)	48.0 (16.1, 74.5)	48.0 (16.1, 74.5)	48.0 (16.1, 74.5)	
Surgery-radio-chemo	100.0 (57.1, 100.0)	40.0 (5.2, 75.3)	20.0 (0.8, 58.2)	20.0 (0.8, 58.2)	
Incomplete	89.3 (76.3, 95.4)	84.5 (70.2, 92.3)	82.0 (67.0, 90.6)	82.0 (67.0, 90.6)	
No treatment	100.0 (62.5, 100.0)	100.0 (62.5, 100.0)	100.0 (62.5, 100.0)	100.0 (62.5, 100.0)	
Anatomical localization					
Supratentorial	98.5 (96.2, 99.5)	95.5 (92.2, 97.4)	93.1 (89.1, 95.7)	88.4 (81.9, 92.7)	0.0068
Infratentorial	98.1 (95.5, 99.2)	96.0 (92.7, 97.8)	93.8 (89.6, 96.3)	93.8 (89.6, 96.3)	
Spinal	100.0 (88.9, 100.0)	87.4 (65.6, 95.8)	72.8 (49.0, 86.9)	72.8 (49.0, 86.9)	
Multiple or unknown	90.9 (50.8, 98.7)	90.9 (50.8, 98.7)	80.8 (42.3, 94.9)	80.8 (42.3, 94.9)	

Data is presented as observed survival (95% confidence interval, 95%CI)

^a ^The *P* values are calculated by log-rank tests

^b ^Consist of neuronal and mixed neuronal-glial tumors

^c ^Consist of oligodendrogliomas, mixed and unspecified gliomas, neuroepithelial glial tumors of uncertain origin

*pLGG* pediatric low-grade gliomas, *Chemo* chemotherapy, *Radio* radiotherapy, *Surgery-Chemo* surgery with chemotherapy, *Surgery-Radio* surgery with radiotherapy, *Surgery-Radio-Chemo* surgery with radiotherapy and chemotherapy

**Table 5:** Adjusted hazard ratios for patient, tumor, and treatment characteristics associated with the composite outcome of death, progression, recurrence, or metastasis

Characteristic	HR	95% CI	*P* value
Age at diagnosis (y)			
0–1	1.00		< 0.0001
2–11	0.50	0.31, 0.81	
12–15	0.25	0.13, 0.47	
16–17	0.65	0.27, 1.60	
Sex			
Female	1.00		0.702
Male	0.94	0.67, 1.30	
Calendar period of diagnosis			
1990–1999	1.00		0.071
2000–2009	0.72	0.47, 1.09	
2010–2019	1.12	0.73, 1.71	
Language region of diagnosis			
German	1.00		0.344
French/Italian	1.20	0.85, 1.70	
Anatomical localization			
Infra	1.00		0.104
Supra	1.20	0.82, 1.75	
Spinal	2.06	1.10, 3.88	
Histological subtype			
Pilocytic astrocytomas	1.00		0.857
Astrocytomas (excluded pilocytic)	0.85	0.29, 2.49	
Other specified and unspecified intracranial and intraspinal neoplasms	0.94	0.57, 1.55	
Other gliomas	0.63	0.19, 2.08	
WHO grade			
Grade 1	1.00		0.615
Grade 2	1.31	0.45, 3.84	
Type of treating hospital			
Non SPOG	1.00		0.003
SPOG	2.62	1.33, 5.18	
Treatment within 1 y of diagnosis			
Surgery only	1.00		< 0.0001
Surgery with other	5.29	3.45, 8.13	
Other ^a^	3.33	1.93, 5.74	
Incomplete or no treatment	2.64	1.48, 4.71	

^a ^Includes radiotherapy, and chemotherapy alone or as a combination treatment

*HR* hazard ratio, *95% CI* 95% confidence interval, *WHO* World Health Organization, *SPOG* Swiss Peadiatric Oncology Group

The different treatment methods during the first year after diagnosis were associated with different OS (Table 4, Fig. 2c). By the end of follow-up, no deaths were observed among the eight children (1.4%) who didn’t receive any treatment within the first year after diagnosis. Children solely treated with surgery had the highest 5- and 10-year OS (99.0% and 97.2%, respectively), whereas children treated with surgery followed by radio- and/or chemotherapy had the lowest 5- and 10-year OS (40% and 20%, respectively). Treatment during the first year after diagnosis appeared to be associated the strongest with EFS, with children being treated with surgery only showing the lowest risk for recurrence, metastasis, progression or death compared to the children that received other treatment methods or combination treatment with surgery (Table 5). The OS appeared to differ depending on the anatomical localization of the pLGG (*P* = 0.007). Tumors located in the spine showed significantly lower 5-,10-year and OS compared to pLGG located elsewhere (Table 4). The 10-year survival rate for supratentorial pLGG was 93.1% (95% CI 89.1%–95.7%), for infratentorial pLGG was 93.8% (95% CI 89.6%–96.3%), for spinal pLGG was 72.8% (95% CI 49.0%–86.9%), and for multifocal or unknown location pLGG was 80.8 (95% CI 42.3%–94.9%). Spinal pLGG also showed a higher risk for recurrence, metastasis, progression, or death compared to infratentorial tumors (adjusted HR = 2.09, 95% CI 1.10–3.97). Nevertheless, there was no evidence for association between anatomical localization and EFS (Table 5, Fig. 2d).

## Discussion

This is the first study on the incidence and survival of children with pLGG in Switzerland. We estimated a total incidence of 1.4 pLGGs per 100,000 children per year during 1990–2019. The histopathological subtype of pLGG appears to influence OS, with pilocytic astrocytoma showing the highest OS rates. Few children had spinal pLGG, which was associated with the lowest OS). Treatment during the first year after diagnosis appeared to influence OS and be the strongest determinant of EFS, a finding most likely biased by tumor characteristics, meaning that more severe tumors might demand exposure to more treatments and have worse outcomes. Three-quarters of the children underwent surgery alone as treatment and showed the highest OS compared to those who required radio- and/or chemotherapy after surgery.

The increased incidence rate over the observed time periods could be due to improved detection rates with increased patients receiving imaging. Further, other factors could be due to the overall higher surgical rates, and more patients being deemed eligible for surgery. The reported demographics in our epidemiological cohort, including age, sex, anatomical location, and histopathology, are comparable to previously reported studies [5, 26–28]. Since the proportion of children with registered underlying diseases was small in our study (13%), we decided to discuss only the presence of NF-1. The *NF-1* mutations were reported for 4% in our study, which is lower compared to other cohorts where it has been reported in up to 10.5% [6]. This difference could be due to underdiagnosis of disease, especially when genetic testing was not as widespread as nowadays, delayed diagnosis in adulthood, or underreporting (underlying diseases have not been systematically collected by the ChCR and were only registered when indicated on the pathology reports of patients). Further, patients with known NF-1 might not undergo biopsy due to very pathognomonic tumors and, as a consequence, might not be registered in the ChCR, which could be contributing to the low NF-1 rate [12].

Our analysis showed that despite the gradual increase in survival over the observed calendar periods, indicating an improvement in the overall care of pLGG, the EFS subsequently decreased over the calendar periods, with the lowest 5-year EFS of 70.9% between 2015 and 2019 compared to 81.2% between 1995 and 2004. This could be due to improved follow-ups and diagnostics, allowing treating physicians to detect even small recurrences. Furthermore, this trend has been shifting towards an intended subtotal resection in eloquent areas to avoid neurological deficits, due to improved adjuvant treatments, which could lead to a higher rate of radiological progression, but can be successfully managed with medication such as chemotherapy [29, 30]. Further, due to less toxic therapies, the interval to treatment might be shorter, given different indications over time.

The high survival rates of over 90% for pLGG have also been reported in other studies from first-world countries [5, 26, 27, 31]. Despite the excellent outcomes in high-income countries, there is a large disparity in middle- and low-income countries [31–33], where survival rates range from approximately 60% to over 90% [31]. Given this disparity around the globe, pLGG is one of the tumors included in the WHO initiative against cancer, aiming to increase survival by 2030 worldwide [34]. Further, it has to be expected that pLGG is underdiagnosed in developing countries, given the limited access to medical resources [31]. Prediction models estimated that 2.9 million childhood cancer cases will be missed between 2015 and 2030, of which a large proportion will include pLGG [35]. Ultimately, one of the key prognostic factors for survival and outcome remains the country of residence when being diagnosed with pLGG [33]. Further, in certain countries, access to more novel treatments such as MEK inhibitors might be limited. Apart from these epidemiological and social challenges, other reported prognostic factors on outcome and survival are histopathology, molecular pathology, and the need for radiation therapy [5].

In our study, children undergoing radiation, either alone or in combination with surgery and/or chemotherapy, comprised only 6% of our population. At the end of our study follow-up, OS after radiation only, after radiation and chemotherapy, and after surgery, radiation, and chemotherapy was 60%, 58%, and 20%, respectively. Comparable results were observed by an analysis of the SEER database including 4040 children diagnosed with pLGG from 1973 to 2008 [5]. Given that our study only focused on including treatment in the first year, we might have recorded lower adjuvant treatment rates, which might have been administered years after initial diagnosis. Further, children requiring radiation have been shown to experience a significantly lower quality of life during long-term follow-up [5]. However, this could also be biased by the fact that children requiring radiation have either a remaining tumor with a less favorable histology or are not amenable to surgery or recurrence, which could influence the OS more than the radiation itself [5].

The pLGG is a heterogeneous group of different tumors. Given our study period (1990–2019), the histopathological subtypes in our study do not follow the newest WHO 2021 guidelines. Further, our data does not include any molecular characteristics of the included pLGG. Molecular information is now included in the WHO classification and is a key determining factor of prognosis. A BRAF aberration is found in up to 70% of all sporadic pLGG and the somatic driver mutation is aberrant signaling of the mitogen-activated protein kinase (MAPK) pathway [32, 36–38]. These molecular aberrations, which have been discovered with increasing precision over the last 1–2 decades, are not only important for classification but also have an impact on prognosis and treatment [7, 37].

We observed in our study that children aged 16–17 years had a lower survival compared to the other age groups (Table 4). This could be because pLGG occurring in late teenage years might resemble adult LGGs, possibly harboring different mutations [5, 12, 39, 40]. A large analysis of mutations in adolescents and young adults showed that only 30% harbored typical rat sarcoma virus (RAS) / MAPK pathway mutations, which were associated with a better outcome than gliomas lacking these molecular mutations [41, 42]. Some patients harbor specific mutations which predispose them to a poorer outcome even without malignant transformation, which is exceedingly rare, and only occurs in approximately 3% of all pLGG [39, 43].

Despite advances in targeted therapies, the mainstay of treatment is still surgical resection, which was also reflected in our cohort and other studies, with 75% of all children undergoing only surgery as their primary treatment, which is comparable to other studies [5, 32, 44, 45]. However, tumors that cannot be safely removed due to their eloquent region, recurrent or multifocal tumors, often require additional treatment. Even in the molecular era, this usually includes chemotherapy as a first adjunct. The first chemotherapy regimens used were a combination of carboplatin or vincristine or multi-agent combinations of thioguanine, procarbazine, chloroethyl-cyclohexyl-nitrosourea (CCNU, lomustine), and vincristine (TPCV regimen). Single-agent vinblastine was then shown as equally effective and less burdensome [6, 32, 46–49] and is, in principle, still the first-line treatment approach for non-*BRAF V600E*-mutated pLGG today. However, given that pLGG with its high OS rates, should be regarded as a chronic disease, the toxicity-induced side effects of these chemotherapeutic agents are not to be underestimated [15, 27]. In our cohort, 7.1% of all children received surgery followed by chemotherapy in their first year of treatment, while 3.2% received upfront chemotherapy alone. The severe long-term side effects, including neurocognitive and endocrine deficiencies in these children, must be considered when recommending adjuvant treatments. Nowadays, it is standard practice that adjuvant drug therapies are only administered if tumors show progression or symptoms, and the associated risk of a bad outcome outweighs the risk of therapy [32]. Additionally, patients with NF-1 have a higher risk of developing secondary radiation-induced neoplasms and should generally be avoided in this population. However, radiation therapy has also undergone modernization over the years, becoming more precise with lower rates of side effects [50]. However, nowadays it is only administered in very rare cases. In Switzerland, children standardly undergo proton beam radiation therapy due to its improved clinical outcomes [51].

Due to the additional molecular and genetic information gained over the last two decades, newer targeted therapies have been established for pLGG. One of the main paradigm changes was the introduction of the MEK inhibitors such as selumetinib or trametinib. With these targeted therapies up to 50% of all children reach a partial response [52]. A recent randomized controlled trial (RCT) comparing MEK/BRAF inhibitors (trametinib plus dabrafenib) versus chemotherapy (carboplatin plus vincristine) in patients with the *BRAF V600E* mutation showed that children in the MEK/BRAF inhibitor group had a significantly higher response rate of 47% compared to 11% in the chemotherapy group, improved quality of life, and a longer progression-free survival of 20.1 months versus 7.4 months [53]. After this trial, the combination of MEK/BRAF inhibitors became the new first-line treatment for *BRAF V600E* mutated pLGG. Given the inclusion period in our cohort, the data regarding the molecular findings was absent, and all patients were diagnosed and treated before the widespread use of MEK and or BRAF inhibitors in pLGG. The main challenges in the use of targeted therapies are the development of resistance and determining the optimal treatment duration, due to the well-known rebound phenomenon after discontinuation of targeted drugs (especially in *BRAF V600E* mutated tumors) [32]. In our cohort, only 1.4% children did not undergo any treatment. This could either be due to very small lesions which were just followed radiographically, or the refusal of therapy. Especially in children with NF-1, radiographic findings can be very pathognomonic, for which surgery did not seem warranted [54, 55]. Overall, 7.1% did not undergo any surgical treatment in their first year. This number is very low compared to a German and a British series, where up to 35.2% did not undergo any surgery [6, 56]. This difference could be partially due to their higher percentage of NF-1 children or different tumor sizes upon presentation given the different healthcare systems or heterogeneity in the different treatment paradigms.

The main strength of our study was that our population was determined by using comprehensive cancer registry data in Switzerland. The ChCR has acquired high completeness since 1995, especially for the age group 0–15 years a 95% completeness can be achieved [19]. Its further benefits from a high-quality long-term follow-up for most registered patients with a lost-to-follow-up rate less than 2%. This study still has several limitations. We did not include any information about molecular diagnostics, and the classification of tumors likely differ in some cases compared to the current WHO classification. Given the lack of any molecular findings, no comparison to the up-to-date treatment guidelines and studies can be drawn. Further, for any midline pLGG, no comment regarding a possible *H3K27* mutation is possible, which might lead to some high-grade gliomas being wrongfully diagnosed as pLGG based on histopathology alone. This might lead to a lower OS for pLGG, excluding pilocytic astrocytoma, than in other studies. We only registered treatment in the first year after diagnosis and hence cannot comment on any further treatment during long-term follow-up. Given the long-term survival, many children might undergo additional treatment years after their initial diagnosis, which might not be reflected in our results. Further, patients with high-risk tumor features may have been exposed to treatments because their risk of progression was higher in the first place. Similarly, patients who experience recurrence or progression within the first year following diagnosis typically require further therapeutic interventions beyond primary surgery. Therefore, poor EFS or increased risk factors for poor overall survival may correlate with greater cumulative treatment exposures. Due to the study interval from 1990–2019, targeted therapies are not listed as a treatment option, which, in the current era, are frequently used in relapsed or refractory pLGG after chemotherapy and in *BRAF V600*-altered pLGG as frontline therapy. Additionally, we do not have any information about the chemotherapy regimens or radiation protocols these children were treated with. The ChCR further does not contain any information about the cognitive or quality of life outcomes of these children, and we cannot make any statement about any possible side effects of their treatment or primary tumor. Moreover, our data did not distinguish between different causes of death in our study; hence do not know if the children succumbed to their primary diagnosis or treatment side effects.

In conclusion, this the first nationwide study in Switzerland to report on the epidemiology of pLGG, and found a total incidence of 1.4 per 100,000 children per year. OS was excellent, with 91% of patients, while 25.8% experienced progression, recurrence, metastasis, or death. Survival was notably lower in 16–17 years group, and the event hazard was significantly higher among 0–1 year group compared to older children. Compared to other histopathological subtypes, children with non-pilocytic astrocytoma had lower survival after the first year of diagnosis. The poorest outcomes were observed in children with spinal tumors and in those who underwent radiation therapy, either alone or in combination with other treatments. Future studies incorporating molecular diagnostics and treatment regimens should be conducted to enhance the understanding of pLGG epidemiology.

## Data Availability

Data is available upon reasonable request from the corresponding author and ChCR and would require special permission from the ChCR.

## Declarations

### AI usage statement

No generative AI was used in the creation of this article.

## References

[1] Diwanji TP, Engelman A, Snider JW, Mohindra P. Epidemiology, diagnosis, and optimal management of glioma in adolescents and young adults. Adolesc Health Med Ther. 2017;8:99–113. 10.2147/AHMT.S53391. PMC5624597 28989289

[2] Ostrom QT, Gittleman H, Truitt G, Boscia A, Kruchko C, Barnholtz-Sloan JS. CBTRUS statistical report: primary brain and other central nervous system tumors diagnosed in the United States in 2011–2015. Neuro Oncol. 2011;20:iv1–86. 10.1093/neuonc/noy131. PMC6129949 30445539

[3] Fangusaro J, Witt O, Hernáiz Driever P, Bag AK, de Blank P, Kadom N, et al. Response assessment in paediatric low-grade glioma: recommendations from the Response Assessment in Pediatric Neuro-Oncology (RAPNO) working group. Lancet Oncol. 2020;21:e305–16. 10.1016/s1470-2045(20)30064-4. 32502457

[4] Lin CT, Riva-Cambrin JK. Management of posterior fossa tumors and hydrocephalus in children: a review. Childs Nerv Syst. 2015;31:1781–9. 10.1007/s00381-015-2781-8. 26351230

[5] Bandopadhayay P, Bergthold G, London WB, Goumnerova LC, Morales La Madrid A, Marcus KJ, et al. Long-term outcome of 4,040 children diagnosed with pediatric low-grade gliomas: an analysis of the Surveillance Epidemiology and End Results (SEER) database. Pediatr Blood Cancer. 2014;61:1173–9. 10.1002/pbc.24958. PMC4657506 24482038

[6] Gnekow AK, Falkenstein F, von Hornstein S, Zwiener I, Berkefeld S, Bison B, et al. Long-term follow-up of the multicenter, multidisciplinary treatment study HIT-LGG-1996 for low-grade glioma in children and adolescents of the German Speaking Society of Pediatric Oncology and Hematology. Neuro Oncol. 1996;14:1265–84. 10.1093/neuonc/nos202. PMC3452343 22942186

[7] Fangusaro J, Onar-Thomas A, Poussaint TY, Wu S, Ligon AH, Lindeman N, et al. Selumetinib in paediatric patients with BRAF-aberrant or neurofibromatosis type 1-associated recurrent, refractory, or progressive low-grade glioma: a multicentre, phase 2 trial. Lancet Oncol. 2019;20:1011–22. 10.1016/S1470-2045(19)30277-3. PMC6628202 31151904

[8] Manoharan N, Choi J, Chordas C, Zimmerman MA, Scully J, Clymer J, et al. Trametinib for the treatment of recurrent/progressive pediatric low-grade glioma. J Neurooncol. 2020;149:253–62. 10.1007/s11060-020-03592-8. 32780261

[9] O’Hare P, Cooney T, de Blank P, Gutmann DH, Kieran M, Milde T, et al. Resistance, rebound, and recurrence regrowth patterns in pediatric low-grade glioma treated by MAPK inhibition: a modified Delphi approach to build international consensus-based definitions-International Pediatric Low-Grade Glioma Coalition. Neuro Oncol. 2024;26:1357–66. 10.1093/neuonc/noae074. PMC11300023 38743009

[10] Roka K, Scheinemann K, Avula S, Maduro JH, Thomale UW, Sehested A, et al. European standard clinical practice recommendations for primary pediatric low-grade gliomas. EJC Paediatr Oncol. 2024;4:100169. 10.1016/j.ejcped.2024.100169.

[11] Acharya S, Liu JF, Tatevossian RG, Chiang J, Qaddoumi I, Gajjar A, et al. Risk stratification in pediatric low-grade glioma and glioneuronal tumor treated with radiation therapy: an integrated clinicopathologic and molecular analysis. Neuro Oncol. 2020;22:1203–13. 10.1093/neuonc/noaa031. PMC7594561 32052049

[12] Greuter L, Guzman R, Soleman J. Pediatric and adult low-grade gliomas: where do the differences lie? Children (Basel). 2021;8:1075. 10.3390/children8111075. PMC8624473 34828788

[13] Krishnatry R, Zhukova N, Guerreiro Stucklin AS, Pole JD, Mistry M, Fried I, et al. Clinical and treatment factors determining long-term outcomes for adult survivors of childhood low-grade glioma: a population-based study. Cancer. 2016;122:1261–9. 10.1002/cncr.29907. 26970559

[14] Hossain MJ, Xiao W, Tayeb M, Khan S. Epidemiology and prognostic factors of pediatric brain tumor survival in the US: evidence from four decades of population data. Cancer Epidemiol. 2021;72:101942. 10.1016/j.canep.2021.101942. PMC8142618 33946020

[15] Armstrong GT, Liu Q, Yasui Y, Huang S, Ness KK, Leisenring W, et al. Long-term outcomes among adult survivors of childhood central nervous system malignancies in the Childhood Cancer Survivor Study. J Natl Cancer Inst. 2009;101:946–58. 10.1093/jnci/djp148. PMC2704230 19535780

[16] Michel G, von der Weid NX, Zwahlen M, Redmond S, Strippoli MF, Kuehni CE. Incidence of childhood cancer in Switzerland: the Swiss childhood cancer registry. Pediatr Blood Cancer. 2008;50:46–51. 10.1002/pbc.21129. 17226849

[17] Cao J, Yan W, Zhan Z, Hong X, Yan H. Epidemiology and risk stratification of low-grade gliomas in the United States, 2004-2019: a competing-risk regression model for survival analysis. Front Oncol. 2023;13:1079597. 10.3389/fonc.2023.1079597. PMC10014976 36937393

[18] Gnekow AK, Kandels D, Pietsch T, Bison B, Warmuth-Metz M, Thomale UW, et al. Doubling recruitment of pediatric low-grade glioma within two decades does not change outcome–report from the German LGG studies. Klin Padiatr. 2021;233:107–22. 10.1055/a-1471-5897. 33971689

[19] Schindler M, Mitter V, Bergstraesser E, Gumy-Pause F, Michel G, Kuehni CE, et al. Death certificate notifications in the Swiss Childhood Cancer Registry: assessing completeness and registration procedures. Swiss Med Wkly. 2015;145:w14225. 10.4414/smw.2015.14225. 26700416

[20] Schindler M, Belle FN, Grotzer MA, von der Weid NX, Kuehni CE, Swiss Paediatric Oncology Group SPOG. Childhood cancer survival in Switzerland (1976-2013): time-trends and predictors. Int J Cancer. 1976;140:62–74. 10.1002/ijc.30434. 27632765

[21] Steliarova-Foucher E, Stiller C, Lacour B, Kaatsch P. International classification of childhood cancer, third edition. Cancer. 2005;103:1457–67. 10.1002/cncr.20910. 15712273

[22] Abawi K, Feller A, Lorez M, Michalopoulou E, Roy E, Spycher B, Weitkunat R. Childhood Cancer Registry. https://www.cancer.bfs.admin.ch, https://www.nacr.ch, and https://www.childhoodcancerregistry.ch. Accessed 24 Nov 2025.

[23] Brenner H, Gefeller O. Deriving more up-todate estimates of long-term patient survival. J Clin Epidemiol. 1997;50:211–6. 10.1016/s0895-4356(97)00280-1. 9120515

[24] R Core Team (2021). R: A language and environment for statistical computing. R Foundation for Statistical Computing, Vienna, Austria. https://www.R-project.org/. Accessed 24 Nov 2025.

[25] Therneau T. A Package for Survival Analysis in R_. R package version 3.7-0. https://CRAN.R-project.org/package=survival. Accessed 24 Nov 2025.

[26] Goebel AM, Gnekow AK, Kandels D, Witt O, Schmidt R, HernáizDriever P. Natural history of pediatric low-grade glioma disease—first multi-state model analysis. J Cancer. 2019;10:6314–26. 10.7150/jca.33463. PMC6856735 31772664

[27] Armstrong GT, Conklin HM, Huang S, Srivastava D, Sanford R, Ellison DW, et al. Survival and long-term health and cognitive outcomes after low-grade glioma. Neuro Oncol. 2011;13:223–34. 10.1093/neuonc/noq178. PMC3064628 21177781

[28] Youland RS, Khwaja SS, Schomas DA, Keating GF, Wetjen NM, Laack NN. Prognostic factors and survival patterns in pediatric low-grade gliomas over 4 decades. J Pediatr Hematol Oncol. 2013;35:197–205. 10.1097/MPH.0b013e3182678bf8. 22983418

[29] Plant-Fox AS, Tabori U. Future perspective of targeted treatments in pediatric low-grade glioma (pLGG): the evolution of standard-of-care and challenges of a new era. Childs Nerv Syst. 2024;40:3291–9. 10.1007/s00381-024-06504-7. 39085626

[30] Kameda-Smith MM, Green K, Hutton DL, Jeelani NUO, Thompson DNP, Hargrave D, et al. The role of reoperation in pediatric cerebellar pilocytic astrocytoma. J Neurosurg Pediatr. 2024;34:169–75. 10.3171/2024.2.peds23236. 38759245

[31] Ward R, Jones HM, Witt D, Boop F, Bouffet E, Rodriguez-Galindo C, et al. Outcomes of children with low-grade gliomas in low- and middle-income countries: a systematic review. JCO Glob Oncol. 2022;8:e2200199. 10.1200/GO.22.00199. PMC9812478 36198134

[32] Fangusaro J, Jones DT, Packer RJ, Gutmann DH, Milde T, Witt O, et al. Pediatric low-grade glioma: state-of-the-art and ongoing challenges. Neuro Oncol. 2024;26:25–37. 10.1093/neuonc/noad195. PMC10768984 37944912

[33] Moreira DC, Bouffet E, Qaddoumi I. The greatest challenge for pediatric low-grade glioma. Neuro Oncol. 2024;26:975–6. 10.1093/neuonc/noae004. PMC11066925 38339843

[34] WHO Global Initiative for Childhood Cancer: An Overview. https://www.who.int/publications/m/item/global-initiative-for-childhood-cancer. Accessed 24 Nov 2025.

[35] Ward ZJ, Yeh JM, Bhakta N, Frazier AL, Atun R. Estimating the total incidence of global childhood cancer: a simulation-based analysis. Lancet Oncol. 2019;20:483–93. 10.1016/S1470-2045(18)30909-4. 30824204

[36] Zhang J, Wu G, Miller CP, Tatevossian RG, Dalton JD, Tang B, et al. Whole-genome sequencing identifies genetic alterations in pediatric low-grade gliomas. Nat Genet. 2013;45:602–12. 10.1038/ng.2611. PMC3727232 23583981

[37] Jones DT, Kocialkowski S, Liu L, Pearson DM, Bäcklund LM, Ichimura K, et al. Tandem duplication producing a novel oncogenic BRAF fusion gene defines the majority of pilocytic astrocytomas. Cancer Res. 2008;68:8673–7. 10.1158/0008-5472.CAN-08-2097. PMC2577184 18974108

[38] Jones DTW, Kieran MW, Bouffet E, Alexandrescu S, Bandopadhayay P, Bornhorst M, et al. Pediatric low-grade gliomas: next biologically driven steps. Neuro Oncol. 2018;20:160–73. 10.1093/neuonc/nox141. PMC5786244 29016845

[39] Lazow MA, Hoffman L, Schafer A, Osorio DS, Boué DR, Rush S, et al. Characterizing temporal genomic heterogeneity in pediatric low-grade gliomas. Acta Neuropathol Commun. 2020;8:182. 10.1186/s40478-020-01054-w. PMC7643477 33153497

[40] Bennett J, Yeo KK, Tabori U, Hawkins C, Lim-Fat MJ. Pediatric-type low-grade gliomas in adolescents and young adults-challenges and emerging paradigms. Childs Nerv Syst. 2024;40:3329–39. 10.1007/s00381-024-06449-x. 38761264

[41] Bennett J, Levine AB, Nobre L, Negm L, Chung J, Fang K, et al. A population-based analysis of the molecular landscape of glioma in adolescents and young adults reveals insights into gliomagenesis. Nat Rev Cancer. 2025;6:1102–19. 10.1038/s43018-025-00962-x. 40335748

[42] Cacciotti C, Tabori U, Hawkins C, Bennett J. Targeting the RAS/MAPK pathway in children with glioma. J Neurooncol. 2025;171:265–77. 10.1007/s11060-024-04857-2. 39448518

[43] Soleman J, Constantini S, Roth J. Incidental brain tumor findings in children: prevalence, natural history, management, controversies, challenges, and dilemmas. Childs Nerv Syst. 2024;40:3179–87. 10.1007/s00381-024-06598-z. PMC11511734 39215810

[44] Wisoff JH, Sanford RA, Heier LA, Sposto R, Burger PC, Yates AJ, et al. Primary neurosurgery for pediatric low-grade gliomas: a prospective multi-institutional study from the Children’s Oncology Group. Neurosurgery. 2011;68:1548–54. 10.1227/NEU.0b013e318214a66e. 21368693

[45] Lorincz KN, Gorodezki D, Schittenhelm J, Zipfel J, Tellermann J, Tatagiba M, et al. Role of surgery in the treatment of pediatric low-grade glioma with various degrees of brain stem involvement. Childs Nerv Syst. 2024;40:3037–50. 10.1007/s00381-024-06561-y. PMC11511697 39145885

[46] Gnekow AK, Kandels D, Tilburg CV, Azizi AA, Opocher E, Stokland T, et al. SIOP-E-BTG and GPOH guidelines for diagnosis and treatment of children and adolescents with low grade glioma. Klin Padiatr. 2019;231:107–35. 10.1055/a-0889-8256. 31108561

[47] Gnekow AK, Walker DA, Kandels D, Picton S, Perilongo Giorgio, Grill J, et al. A European randomised controlled trial of the addition of etoposide to standard vincristine and carboplatin induction as part of an 18-month treatment programme for childhood (≤ 16 years) low grade glioma a final report. Eur J Cancer. 2017;81:206–25. 10.1016/j.ejca.2017.04.019. PMC5517338 28649001

[48] Packer RJ, Lange B, Ater J, Nicholson HS, Allen J, Walker R, et al. Carboplatin and vincristine for recurrent and newly diagnosed low-grade gliomas of childhood. J Clin Oncol. 1993;11:850–6. 10.1200/JCO.1993.11.5.850. 8487049

[49] Buckner JC, Shaw EG, Pugh SL, Chakravarti A, Gilbert MR, Barger GR, et al. Radiation plus procarbazine, CCNU, and vincristine in low-grade glioma. N Engl J Med. 2016;374:1344–55. 10.1056/NEJMoa1500925. PMC5170873 27050206

[50] Bitterman DS, MacDonald SM, Yock TI, Tarbell NJ, Wright KD, Chi SN, et al. Revisiting the role of radiation therapy for pediatric low-grade glioma. J Clin Oncol. 2019;37:3335–9. 10.1200/JCO.19.01270. PMC9798905 31498029

[51] Tran S, Lim PS, Bojaxhiu B, Teske C, Baust K, Zepter S, et al. Clinical outcomes and quality of life in children and adolescents with primary brain tumors treated with pencil beam scanning proton therapy. Pediatr Blood Cancer. 2020;67:e28465. 10.1002/pbc.28465. 32902137

[52] Sigaud R, Albert TK, Hess C, Hielscher T, Winkler N, Kocher D, et al. MAPK inhibitor sensitivity scores predict sensitivity driven by the immune infiltration in pediatric low-grade gliomas. Nat Commun. 2023;14:4533. 10.1038/s41467-023-40235-8. PMC10374577 37500667

[53] Bouffet E, Hansford JR, Garrè ML, Hara J, Plant-Fox A, Aerts I, et al. Dabrafenib plus trametinib in pediatric glioma with BRAF V600 mutations. N Engl J Med. 2023;389:1108–20. 10.1056/NEJMoa2303815. 37733309

[54] Soleman J, Kozyrev DA, Ben-Sira L, Constantini S, Roth J. Management of incidental brain tumors in children: a systematic review. Childs Nerv Syst. 2020;36:1607–19. 10.1007/s00381-020-04658-8. 32377829

[55] Roth J, Soleman J, Paraskevopoulos D, Keating RF, Constantini S. Incidental brain tumors in children: an international neurosurgical, oncological survey. Childs Nerv Syst. 2018;34:1325–33. 10.1007/s00381-018-3836-4. 29802595

[56] Stokland T, Liu JF, Ironside JW, Ellison DW, Taylor R, Robinson KJ, et al. A multivariate analysis of factors determining tumor progression in childhood low-grade glioma: a population-based cohort study (CCLG CNS9702). Neuro Oncol. 2010;12:1257–68. 10.1093/neuonc/noq092. PMC3018938 20861086

